# The Safeguarding Microglia: Central Role for P2Y_12_ Receptors

**DOI:** 10.3389/fphar.2020.627760

**Published:** 2021-01-14

**Authors:** Si-Si Lin, Yong Tang, Peter Illes, Alexei Verkhratsky

**Affiliations:** ^1^ Acupuncture and Tuina School, Chengdu University of Traditional Chinese Medicine, Chengdu, China; ^2^ International Collaborative Center on Big Science Plan for Purine Signalling, Chengdu University of Traditional Chinese Medicine, Chengdu, China; ^3^ Rudolf Boehm Institute for Pharmacology and Toxicology, University of Leipzig, Leipzig, Germany; ^4^ Faculty of Biology, Medicine and Health, The University of Manchester, Manchester, United Kingdom; ^5^ Achucarro Centre for Neuroscience, IKERBASQUE, Basque Foundation for Science, Bilbao, Spain

**Keywords:** microglia, P2Y_12_ receptors, neurone-microglial crosstalk, purinergic signalling, neuroprotective

## Introduction

The brain is the most complex organ of human body composed of several highly specialised and heterogeneous population of cells, represented by neurones, neuroglia (astrocytes, microglia, oligodendrocytes) and cells of brain vasculature. Neurones and neuroglia form neural circuits; different types of glial cells contribute to shaping and maintaining synaptic connections, plasticity, homeostasis, and network level activity through dynamic monitoring and alteration of central nervous system (CNS) functional architecture (Kettenmann et al., 2013; Allen and Lyons, 2018; Verkhratsky and Nedergaard, 2018; Augusto-Oliveira et al., 2020). Microglial cells are scions of foetal macrophages invading the neural tube early in embryonic development (Ginhoux et al., 2013); after settling in the nervous tissue these cells undergo the most remarkable metamorphoses acquiring specific morphology (small soma with long, ramified motile processes) and physiology. In particular, microglial cells gain receptors to neurotransmitters and neuromodulators, while retaining the pattern recognition receptors from their immune heritage; this extended complement of receptors makes microglia arguably the most “receptive” cells in the CNS (Kettenmann et al., 2011; Garaschuk and Verkhratsky, 2019). Among these many receptors, microglia possess several types of purinoceptors, which are linked to microglial housekeeping, neuroprotective and defensive capabilities (Verkhratsky et al., 2009; Tozaki-Saitoh et al., 2012). Purinergic signalling emerges as the key mechanism in the dynamic interactions between neurones and glial cells, with ATP being a classical neurotransmitter and a danger signal damage-associated molecular pattern (DAMP). This duality makes ATP and related purines versatile signalling molecules controlling microglial behaviours in both physiological and pathological context (Domercq et al., 2013; Illes et al., 2020).

The metabotropic P2Y_12_ purinoceptor is of a particular relevance for microglia. First and foremost, the expression of this receptor distinguishes CNS resident microglia from peripheral macrophages (Sasaki et al., 2003; Haynes et al., 2006). Second, in the healthy brain P2Y_12_ receptors are universally and specifically expressed in microglia in all brain regions and across different species from rodents to humans (Sasaki et al., 2003; Mildner et al., 2017); the P2Y_12_ receptors are widely considered to be a signature of microglia in the healthy brain (Hickman et al., 2013; Bosco et al., 2018; Peng et al., 2019). Third, expression of P2Y_12_ receptors is stable from foetal state and throughout human lifespan (Crain et al., 2009; Mildner et al., 2017). The P2Y_12_ receptors share the seven-transmembrane topology characteristic for G-protein coupled receptors of P2Y family (Burnstock and Verkhratsky, 2012). The preferred agonist for P2Y_12_ receptors is adenosine diphosphate (ADP), which in the periphery acts as a major instigator of platelet aggregation and granule secretion thus supporting thrombogenesis (Liverani et al., 2014). In the CNS, microglial P2Y_12_ receptors are activated by ADP deriving from enzymatic degradation of ATP released from neurones, astrocytes and oligodendroglia during their physiological activity or following tissue damage (Abbracchio et al., 2009; Zimmermann et al., 2012). Metabotropic P2Y_12_ receptors are localised in the processes and in the somata of surveilling microglia, where they mediate various aspects of intercellular signalling targeting microglia (Table 1 and Posfai et al., 2019; Vainchtein and Molofsky, 2020).

**TABLE 1:** Microglial P2Y_12_ receptors in healthy and diseased brain.

Specie/age/brain region	Experimental techniques	Main findings	References
Animal models			
Mice/45–180 days/hippocampus Rat/2 weeks	*In vivo* two-photon imaging; immunofluorescence; rat primary microglia live imaging; western blot	Activation of P2Y_12_ receptors triggers extension of microglial processes	Bernier et al. (2019)
Rats/neonatal/forebrain	Real time RT-PCR; calcium imaging; western blot; immunocytochemistry	Stimulation of P2Y_12_ receptors instigated processes extension towards the source of ADP	Tozaki-Saitoh et al. (2017)
Mice/P1/cortex	Immunocytochemistry; IB4 staining; quantitative PCR; western blot	P2Y_12_ receptors-mediated Ca^2+^ signalling regulate the migration and phagocytic ability of microglia during post-natal brain development	Sunkaria et al. (2016)
Mice/P21–P23/hippocampus	Primary microglia culture; *in vitro* phagocytosis assay; calcium imaging; FACS sorting; gene expression arrays; real-time qPCR	Genetic deletion of P2Y_12_ receptors affected microglial phagocytosis and neurogenesis suggesting active role of microglia in regulation of this process	Diaz-Aparicio et al. (2020)
Mice/ventral hippocampus CA1	Constitutive and induced microglia-specific knockout of P2Y_12_ receptors; behaviour tests (open field, elevated plus maze, light/dark box, fear conditioning); *in vivo* two-photon imaging; electrophysiology; immunocytochemistry	P2Y_12_ receptors contribute to microglia-dependent suppression of neuronal excitability as well as to innate fear behaviours	Peng et al. (2019)
Mice/6–8 weeks/somatosensory cortex	Photothrombotic stroke; two-photon imaging; immunocytochemistry and confocal imaging	Expression of P2Y_12_ receptors declined significantly 14 days after stroke; which correlated with the development of secondary neurodegeneration and neuronal damage	Kluge et al. (2019)
Rats/neonatal/cerebral cortex	Facial nerve axotomy; primary cell culture; northern blot; *in situ* hybridisation; immunocytochemistry	P2Y_12_ receptors are expressed selectively in microglia. Number of P2Y_12_ receptor expressing cells increased following facial nerve axotomy	Sasaki et al. (2003)
Mice/12–14 weeks/cortex	Electrophysiology; immunofluorescence; STORM super-resolution microscopy	Spreading depolarisation increased the density of microglial P2Y_12_ receptors and increased association of microglial processes to neurones in P2Y_12_-dependent manner	Varga et al. (2020)
Mice/5, 12 weeks/hippocampus dentate gyrus	Sleep deprivation; behavioural tests (open field test; novel object recognition test; elevated plus maze test); histological examinations; RT-PCR; immunocytochemistry; western blot	Sleep deprivation resulted in a decrease in microglial P2Y_12_ receptors	Tuan and Lee (2019)
Rat/prelimbic cortex, central amygdala, perifornical lateral hypothalamic area, and dorsal raphe nucleus	Immunocytochemistry; densitometry and cell counts; histology; qRT-PCR	Sleep deprivation increased Iba1 staining, but did not affect immunoreactivity of P2Y_12_ receptors and pro-inflammatory cytokines	Hall et al. (2020)
Humans, tissues and cells			
Humans/23–92 years/post-mortem tissue of MDD patients (5) and mentally healthy controls (5)/frontal lobe, temporal lobe, thalamus, subventricular zone	Freshly isolated microglial cell suspension; purified with CD11b^−^ assisted multiplexed single-cell mass cytometry. Immunocytochemistry	A subpopulation of microglia from MDD brains have increased expression of P2Y_12_ receptors, arguably associated with an increase in homeostatic and neuroprotective capacity of microglial cells in the diseased nervous tissue	Bottcher et al. (2020)
Humans/recent-onset schizophrenia patients (20) and 20 non-psychiatric controls (20)/myeloid cell	Monocytes induced into microglia-like cells; RNA isolation and sequencing; mass cytometry; phagocytosis assay; immunocytochemistry and microscopy	P2Y_12_ receptors mRNA was enriched in a sub-population of cells from schizophrenia patients	Ormel et al. (2020)
Humans/dermal fibroblast cells	Human induced pluripotent stem cells (iPSCs); immunocytochemistry; scanning electron microscopy; flow cytometry; engraftment assays electrophysiology; PCR	Microglia derived from iPSCs displayed ramified morphology and 100% expression of P2Y_12_ receptors. Stimulation of these cells with lipopolysaccharide resulted in downregulation of P2Y_12_ receptors expression	Banerjee et al. (2020)
Humans/60–80 years old/occipitalcortex, corpus callosum, choroid plexus	Freshly isolated microglial cell; quantitative RT-PCR; IRF8 + isolation and sorting of nuclei; immunocytochemistry; western blot analysis; flow cytometry	P2Y_12_ receptor expression is unaltered in normal-appearing tissue from MS patients indicating overall preservation of microglia homeostatic phenotype	van der Poel et al. (2019)
Human patients with AD/70–90 years old	Immunocytochemistry, confocal microscopy	The P2Y_12_ positive microglial cells of heterogeneous morphology populated outer regions of senile plaques	Walker et al. (2020)
Human patients with MS/rats (8–11 weeks)/tissue	Experimental autoimmune encephalomyelitis in rats; human microglia isolation; immunocytochemistry; q-PCR; western blot; autoradiography	P2Y_12_ receptors were associated with an anti-inflammatory phenotype; expression of P2Y_12_ receptors was decreased in tissues with active MS lesions	Beaino et al. (2017)
Humans/newborns (5), children (4), adults (5), elderly individuals (5)/cortex, hippocampus	Immunocytochemistry; microscopy	Expression of P2Y_12_ receptors in the brain microglia is stable throughout human lifespan. Density of P2Y_12_ expressing microglia is similarly constant throughout life. CNS pathologies are associated with a decrease in P2Y_12_ immunoreactivity	Mildner et al. (2017)
Human/foetal brain tissue	Human monocyte-derived macrophages culture; immunocytochemistry; quantitative real time PCR; flow cytometry; calcium imaging; cell migration assays; ELISA	P2Y_12_ is selectively expressed on human microglia and elevated under neuropathological conditions that promote Th2 responses, such as parasitic CNS infection	Moore et al. (2015)
Humans/59–78 years old/MCA area mice/12–18 weeks/	MCAO; histology; cloning; *in utero* electroporation; *in vivo* two-photon imaging; calcium imaging; immunocytochemistry; STORM super-resolution imaging; immunoelectrone microscopy; electron tomography	P2Y_12_ receptors support formation and maintenance of somatic microglia-neurone junctions and mediate microglial neuroprotection in ischaemia	Cserep et al. (2020)
Humans/59–78 years old/Mice/8–12 weeks/hypothalamic paraventricular nucleus	*In vivo* pharmacological treatments and chemogenetics; histology; cloning*; in utero* electroporation; isolation of microglial cells; quantification of ATP; *in vivo* two-photon imaging; immunocytochemistry; confocal laser scanning microscopy	Microglial P2Y_12_ receptors are instrumental in defence against neurotropic viruses	Fekete et al. (2018)
Human/30–97 years old/white matter	Post-mortem immunocytochemistry	Activated microglia in the active and slowly expanding lesion sites in the white matter of MS patients demonstrated significant down-regulation of P2Y_12_ receptors, in the inactive lesions however the P2Y_12_ positive microglia re-emerged	Zrzavy et al. (2017)

## Modes of Microglial Patrolling of the Healthy CNS: Role for P2Y_12_ Receptors

Microglial cells are indefatigable surveillants and overseers of the nervous tissue; their ramified processes are in constant move scanning CNS parenchyma (Davalos et al., 2005; Nimmerjahn et al., 2005) with a particular attention paid to neurones (Wake et al., 2009; Cserep et al., 2020). Microglial surveillance of the nervous tissue occurs in several distinct modes.

### Microglia-Dendritic/Synaptic Patrolling

In the healthy brain microglial processes are constantly contacting synaptic contacts located on neuronal dendrites. These microglia-dendritic contacts are instrumental for synaptic pruning in early development, which removes silent, aberrant or redundant synapses by *en passant* phagocytosis (Sierra et al., 2010) thus contributing to shaping neuronal ensembles and supporting neuroplasticity (Kettenmann et al., 2013; Sakai, 2020). Synaptic pruning is controlled by neuronal complement system (Stevens et al., 2007; Schafer et al., 2012), which tags the synapses to be removed, and by neurone-derived chemokine CX3CL1 also known as fractalkine. Microglial cells specifically express fractalkine receptors, activation of which stimulates synaptic pruning by physiological phagocytosis (Paolicelli et al., 2011). At later developmental stages microglia can remove not only whole synapses but also synaptic fragments through the process known as trogocytosis (Weinhard et al., 2018).

Microglia-dendritic interactions are regulated by neuronal activity: an increase in neuronal firing increases the frequency and number of contacts between microglial processes and synapses (Li et al., 2013). Plastic remodelling of the nervous tissue involves substantial changes in microglial morphology, manifested in hyper-ramification of processes, decreased intrinsic motility of processes and increased number of contacts with synaptic sites. P2Y_12_ receptors play a primary role in these preocesses; pharmacological and genetic occlusion of these receptors suppressed both microglial changes and neuronal plasticity, thus revealing contribution of microglia to experience-induced reshaping of neuronal networks (Sipe et al., 2016).

### Microglia*-*Somatic Patrolling

The second distinct type of microglial patrolling is aimed at neuronal somata. In the cortex microglial processes frequently contact neuronal cell bodies. The microglia process-neuronal somata contacts (defined as somatic microglial junctions) last for tens of minutes and even up to 1 h, which is much longer compared to microglia-dendritic or microglia-synaptic contacts which usually last for several minutes only (Cserep et al., 2020). Neuronal part of microglia-somatic junction contains mitochondria and secretory vesicles closely associated with plasmalemma; the microglial part of the junction was characterised by exceptionally high density of P2Y_12_ receptors. The P2Y_12_ receptors control formation of microglia-somatic junctions, as pharmacological blockade of these receptors halves the duration of microglia-somatic contacts. The microglia-somatic junctions seem to be particularly important for neuroprotection after ischemic attack: the stroke greatly increases microglial coverage of neuronal cell bodies; this increase requires operational P2Y_12_ receptors. Inhibition of P2Y_12_-mediated signalling negatively impacts on neurones, which experience greater calcium load and increased functional disconnection. Signalling between neurones and microglial processes at the somatic level is supported by neuronal mitochondria and ATP exocytosis from vesicular-nucleotide transporter (VNUT)-containing secretory vesicles: disruption of either impairs the microglia-somatic junction (Cserep et al., 2020). To summarise, microglial P2Y_12_ receptors provide for specialised interaction between neuronal cell bodies and microglial cells, interaction which appears to be critical for neuroprotection.

### Microglia-Axonal Patrolling

Microglial processes establish intimate contacts with axon initial segments early in development and these contacts are maintained through adulthood probably supporting axonal structure (Baalman et al., 2015). Increased firing of the axon, reflective of neuronal hyperexcitability initiates further extension of microglial processes, which enwrap the axon and suppress axonal action potential generation, thus preventing excitotoxicity. Inhibition of microglial motility blocks this mechanism and facilitates neuronal death (Kato et al., 2016). Which microglial receptors are responsible for axonal patrolling remains unknown, although the involvement of fractalkine receptors has been excluded (Baalman et al., 2015).

### Microglial Processes Converging Response—Counteracting Acute Lesions to the Nervous Tissue

Another type of microglial patrolling is associated with rapid convergence processes response, in which microglial processes swiftly move towards the site of potential injury. Thus response is regulated solely by P2Y_12_ receptors that detect the source of ATP/ADP as a potential damage signal (Davalos et al., 2005). The converging response of microglial processes represents a specific form of patrolling associated with primary defensive function of microglia. This response occurs at the initial stages of various neuropathologies. In particular, local cortical damage, associated with rapid increase in ATP/ADP instantly triggers microglial processes convergence towards the site of the lesion (Haynes et al., 2006). This directional extension of microglial processes involved activation of β1 integrin signalling cascade (Ohsawa et al., 2010). Microglial processes converge on axons after traumatic brain injury to reduce neuronal excitability (Benusa and Lafrenaye, 2020). Similarly, microglial processes move to and enwrap neurones and axons in experimental epilepsy, which again counteracts hyperexcitability and potentially limits the seizures (Eyo et al., 2014). Mechanistically, excessive neuronal activity results in activation of NMDA receptors, which trigger ATP release that translates, through activation of P2Y_12_ receptors, into converging microglial processes response (Dissing-Olesen et al., 2014) Genetic or pharmacological silencing of P2Y_12_ receptors obliterates microglial processes converging response in all these pathological contexts (Haynes et al., 2006; Eyo et al., 2014).

## Microglial P2Y_12_ Receptors in Neurological Diseases

Pathological insults to the CNS invariably stimulate and recruit microglia (Kettenmann et al., 2011; Savage et al., 2019), triggering reactive microgliosis (the commonly used term “activation” is somewhat misleading; microglial cells are activated by numerous signals in physiological context, whereas microgliosis represent response to pathology and hence should be defined as reactivity). Purines and ATP are, as alluded earlier, classic damage-associated molecular patters (DAMP) conserved throughout the evolution (Verkhratsky and Burnstock, 2014). The P2Y_12_ receptor is intimately involved in the early stages of microglial response to the lesion, as discussed in previous chapter, and to the early stages of microgliotic response (Table 1). Stimulation of microglial P2Y_12_ receptors triggers microgliotic transformation into various reactive phenotypes that ultimately climaxes in amoeboid phagocyting microglia (Hanisch and Kettenmann, 2007; Savage et al., 2019). Genetic deletion of P2Y_12_ receptors results deficits in up-regulation of K^+^ outward rectifying channels and in membrane ruffling and chemotaxis of amoeboid microglia (Swiatkowski et al., 2016).

Reactive microgliosis however almost invariably results in down-regulation of expression of microglial P2Y_12_ receptors (Zrzavy et al., 2017). Injection of LPS into the striatum triggers massive activation of microglial cells associated with almost complete disappearance of P2Y_12_ receptors 4 days after the insult (Fukumoto et al., 2019); treatment of human induced pluripotent stem cells derived microglia with LPS likewise resulted in disappearance of P2Y_12_ receptors (Banerjee et al., 2020). Similarly, experimental stroke induced gradual and almost compete disappearance of microglial P2Y_12_ receptors (Kluge et al., 2019); down-regulation of P2Y_12_ receptors have been observed in microglia in several chronic neurological diseases (Mildner et al., 2017; Zrzavy et al., 2017). Recent investigations however have found P2Y_12_ receptors expression in microglia in several chronic neurological and neuropsychiatric conditions. The P2Y_12_-positive microglial cells were detected in the microglia freshly isolated from post-mortem brains of human patients suffering from major depressive disorder (Bottcher et al., 2020). Similarly microglia bearing P2Y_12_ receptors were found in the outer regions of senile plaques in post-mortem tissues from Alzheimer’s disease patients (Walker et al., 2020). These results indicate that P2Y_12_ microglia populate diseased brains, which might be associated with rise of defensive, safeguarding microglial phenotypes, distinct from reactive microglia.

## Conclusion

The P2Y_12_ purinoceptors are signature receptors of microglia in the healthy brain. These receptors mediate patrolling behaviours of surveilling microglia and coordinate neuronal activity with operation of microglia. The P2Y_12_ receptors are instrumental for microglial response to neuropathological lesion, and are responsible for the initiation of reactive microgliosis. Reactive microglia as a rule do not express P2Y_12_ receptors, however in neurodegenerative and neuropsychiatric disease the population of P2Y_12_-bearing microglia (distinct form reactive microglia) remains; these cells arguably participate in defensive, safeguarding responses against neuropathology.

## Author Contributions

Conceptualisation: S-SL and AV; writing—original draft preparation: S-SL and AV; literature review, writing—review and editing: S-SL, YT, PI, and AV.

## Funding

Our work was supported by National Key R&D Program of China (2019YFC1709101), the Project First-Class Disciplines Development of Chengdu University of Traditional Chinese Medicine (CZYHW1901), the National Natural Science Foundation of China (81774437 and 81973969), and Science and Technology Program of Sichuan Province, China (2019YFH0108).

## Conflict of Interest

The authors declare that the research was conducted in the absence of any commercial or financial relationships that could be construed as a potential conflict of interest.
